# Identification of a New Delhi metallo-β-lactamase-4 (NDM-4)-producing *Escherichia coli* in Italy

**DOI:** 10.1186/1471-2180-14-148

**Published:** 2014-06-07

**Authors:** Erika Coppo, Valerio Del Bono, Francesco Ventura, Marco Camera, Giovanni Orengo, Claudio Viscoli, Anna Marchese

**Affiliations:** 1 Microbiology Unit, DISC Department, University of Genoa, Largo R. Benzi 10, 16132 Genoa, Italy; 2 Infectious Diseases Unit, IRCCS AOU San Martino-IST, Largo R. Benzi 10, 16132 Genoa, Italy; 3 Rehabilitation Unit, IRCCS AOU San Martino-IST, Largo R. Benzi 10, 16132 Genoa, Italy; 4 Hygiene and Epidemiology Unit, IRCCS AOU San Martino-IST, Largo R. Benzi 10, 16132 Genoa, Italy; 5 Infectious Diseases Unit DISSAL, University of Genoa, IRCCS AOU San Martino-IST, Largo R. Benzi 10, 16132 Genoa, Italy; 6 Microbiology Unit DISC, University of Genoa, IRCCS AOU San Martino-IST, Largo R. Benzi 10, 16132 Genoa, Italy

**Keywords:** Carbapenemases, *E.coli*, ST405, Class 1 integron

## Abstract

**Background:**

During June-July 2012, six imipenem-resistant *Escherichia coli* isolates were isolated from two patients hospitalized in a ward of one large tertiary-care hospital in Genoa, Italy. Genetic features associated with *bla*_NDM-4_ gene were investigated.

**Results:**

The isolates exhibited the same PFGE profile and a multidrug-resistant (MDR) phenotype to aminoglycosides, fluoroquinolones, and β-lactams. The strains produced the NDM-4 carbapenemase and the *bla*_NDM-4_ gene was part of the variable region of a class 1 integron. MLST analysis revealed that all isolates belonged to sequence type 405 (ST405).

**Conclusions:**

This is the first report on the emergence of an MDR strain of *E.coli* producing the NDM-4 MBL in Italy.

## Background

The emerging New Delhi metallo-β-lactamase (NDM), an acquired class B carbapenemase that was first detected in *Klebsiella pneumoniae* isolate from a Swedish patient of Indian origin has become a major public health concern worldwide [1].

Two cases of the new variant, NDM-4, have been recently described in isolated recovered from patients previously hospitalized in India and Cameroon [2,3]. In Italy, a few cases of NDM-1 producing *E.coli* and *K.pneumoniae* have been reported during 2009 and 2011 [4,5].

In this study we described six NDM-4-producing *E.coli* isolates obtained from two patients admitted to an Italian hospital. We also present data on the localization and the genetic environment of the *bla*_NDM-4_ gene.

## Methods

### Bacterial strains

Six *E.coli* isolated from urine samples of two inpatients at the San Martino-IST University Hospital (Genoa, Italy) were studied. Isolates were taken as part of standard patient care and informed consent for the use of clinical data has been obtained by both patients.

### Strain identification, antibiotic susceptibility testing and phenotypic screening for MBL production

Routine identification and antibiotic susceptibility testing were carried out using the Vitek-2 automated system (BioMérieux, Marcy-L’etoile, France). *In vitro* activity of carbapenems, aztreonam, fosfomycin and nitrofurantoin was further determined by the broth microdilution method and interpreted according to the of European Committee on Antimicrobial Susceptibility Testing (EUCAST ) guidelines (Version 4.0, 2014) [6]. To detect metallo-β-lactamase (MBL) production, a synergy test using imipenem and EDTA discs was used [7].

### Pulsed-field gel electrophoresis (PFGE)

Genomic DNA was prepared, digested with *XbaI* (New England Biolabs Inc., MA, USA) and subjected to PFGE with the CHEF DRII device (Bio-rad, Milan, Italy), as described previously [8]. Fingerprinting pattern was interpreted according to the method of Tenover *et al*. [9].

### Multilocus sequence typing (MLST)

MLST was carried out using protocols and conditions described on the *E.coli* MLST website (http://mlst.warwick.ac.uk/mlst/dbs/Ecoli/documents/primersColi_html). Sequence types were assigned using the website interface.

### Molecular analysis techniques

Polymerase chain reaction (PCR) amplification of the *bla*_NDM_ gene and direct sequencing of the PCR products was performed as previously described [10]. Screening for resistance genes was carried out using primers and conditions previously described [11-13]. Phylogenetic analysis using multiplex PCR method as described previously [14] was used. PCR experiments were performed to identify the upstream- and downstream-located regions of the *bla*_NDM-4_ gene [15]. Mapping of the variable region of class 1 integron was performed by PCR as described previously [16]. The genetic environment of *bla*_NDM-4_ was studied by PCR mapping and sequencing as described previously [13].

### Conjugation assay *and plasmid study*

Plasmid transfer was attempted by conjugation, using *E.coli* J53 as the recipient, as described previously [17]. Plasmid DNA, isolated from *E.coli*, was obtained by the alkaline lysis method and was used as a template in PCR analysis with primers that are specific for *bla*_NDM_ and *bla*_CTX-M_[18]. To rule out chromosomal DNA contamination the template was used to amplify an internal fragment of the house-keeping *recA* gene. A PCR-based replicon typing method was used to identify the incompatibility group [19].

## Results

### Bacteria and patients

**Figure 1:**
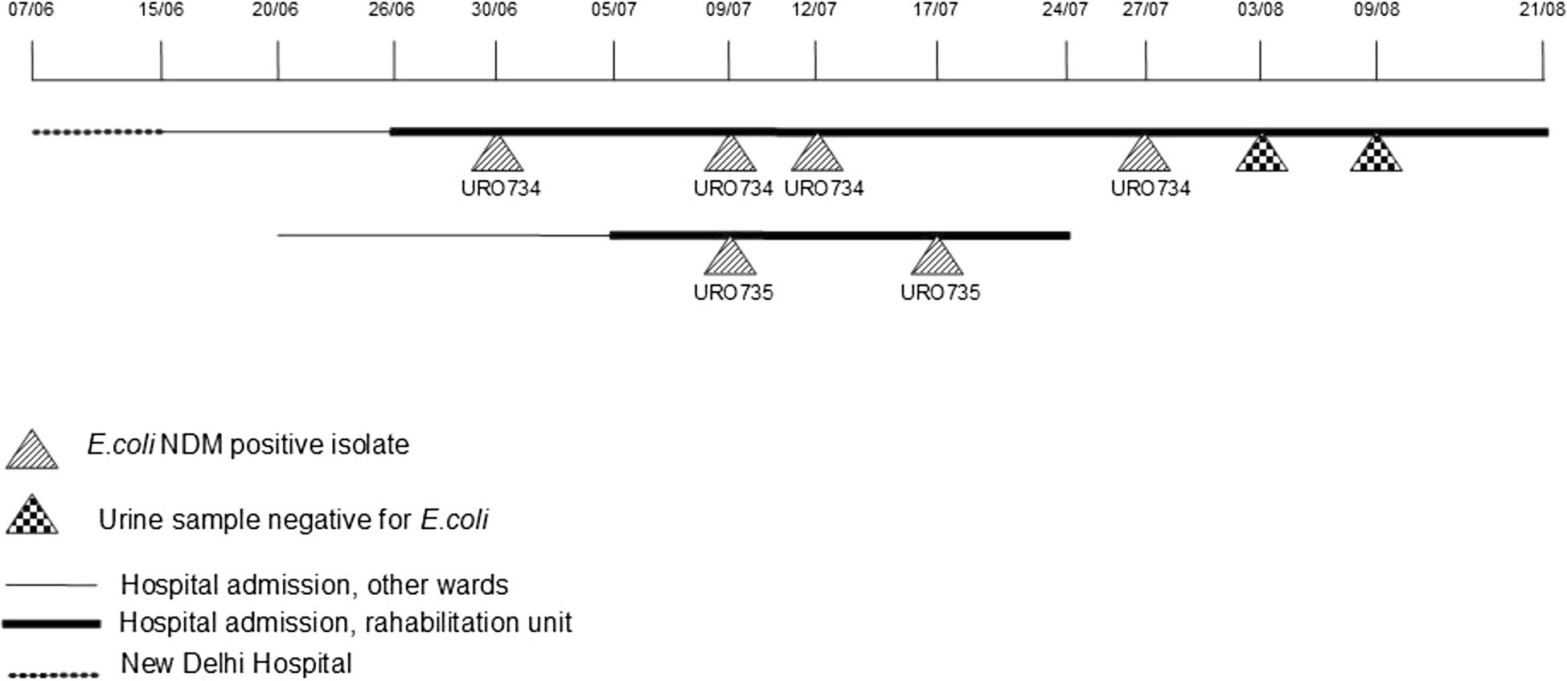
**Time of isolation of NDM-4 positive *E.coli *** **from patient 1 and 2.**

The first NDM-4-positive *E.coli* isolate (URO734, index strain) was detected from the urine of a 61-year-old male inpatient (patient 1) of the rehabilitation unit of the San Martino-IST Hospital on 30 June 2012 (Figure 1). At the beginning of June, the patient was hospitalized for 7 days, in a hospital in New Delhi, India, with a history of right middle cerebral artery ischemic stroke and left-sided hemiparesis. On 15 June 2012 the patient was admitted to San Martino-IST stroke center and on 26 June he was transferred in the rehabilitation unit for 57 days. Subsequent urine samples, collected during the hospitalization period (9 July, 12 July, 27 July), continued to yield NDM-4-positive *E. coli* showing the same MDR phenotype as URO734 until 27 July. The patient was empirically treated with colistin. Subsequent urine samples (03 August, 09 August) were negative for *E. coli*.

A second case of urinary tract infection sustained by NDM-4-positive *E. coli* was detected in July 2012 in another inpatient (patient 2), a 79-year-old man, with a history of hip replacement, who was admitted to the same rehabilitation unit during a period overlapping the admittance of the index case. The first isolate from patient 2 (isolate URO735) was contemporary with the second isolate from patient 1. Subsequent urine sample, collected during the admission period (17 July), continued to yield NDM-4-positive *E. coli*, showing the same MDR phenotype as URO734. Initially, the patient was empirically treated with pipemidic acid and then, after antimicrobial susceptibility results were available, with nitrofurantoin. The clinical condition of the patient improved and the patient was discharged, without further positive urine culture. No history of travel in India or other NDM endemic areas was reported for this patient.

### Antimicrobial susceptibility

The NDM-4-positive *E. coli* isolates exhibited a MDR phenotype to aminoglycosides, fluoroquinolones, and all β-lactams tested. The strains were susceptible to colistin, nitrofurantoin, fosfomycin and tigecycline (Table 1). All NDM-4-positive isolates produced metallo-β-lactamase (MBL) activity by the imipenem-EDTA double-disk synergy test.

**Table 1:** **Minimum Inhibitory Concentrations of selected antimicrobials agents against NDM-4-producing *E.coli *** **isolates**

**Isolates**	**Patients**	**Sample collection date**	**Sample**	**MIC (μg\ml)**													
				**AK**	**AMC**	**CAZ**	**LEV**	**CO**	**IPM**	**MER**	**GM**	**TYG**	**SXT**	**CTX**	**NIT**	**FOS**	**AZT**
URO734	1	30 Jun	Urine	>64	>32	>64	>8	<0,5	16	>128	>16	<0,5	>320	>64	64	<2	>64
URO734	1	09 Jul	Urine	>64	>32	>64	>8	<0,5	16	>128	>16	<0,5	>320	>64	64	<2	>64
URO734	1	12 Jul	Urine	>64	>32	>64	>8	<0,5	16	>128	>16	<0,5	>320	>64	64	<2	>64
URO734	1	27 Jul	Urine	>64	>32	>64	>8	<0,5	16	>128	>16	<0,5	>320	>64	64	<2	>64
URO735	2	09 Jul	Urine	>64	>32	>64	>8	<0,5	16	>128	>16	<0,5	>320	>64	64	<2	>64
URO735	2	17 Jul	Urine	>64	>32	>64	>8	<0,5	16	>128	>16	<0,5	>320	>64	64	<2	>64

*AK*: amikacin; *AMC*: amoxicillin/clavulanic acid; *CAZ*: ceftazidime; *LEV*: levofloxacin; *CO*: colistin; *IPM*: imipenem; *MER*: meropenem; *GM*: gentamicin; *TYG*: tigecycline; *SXT*: thrimethoprim/sulfamethoxazole; *CTX*: cefotaxime; *NIT*: nitrofurantoin; *FOS*: fosfomycin, *AZT*: aztreonam.

### Phylogenetic group and PFGE

**Figure 2:**
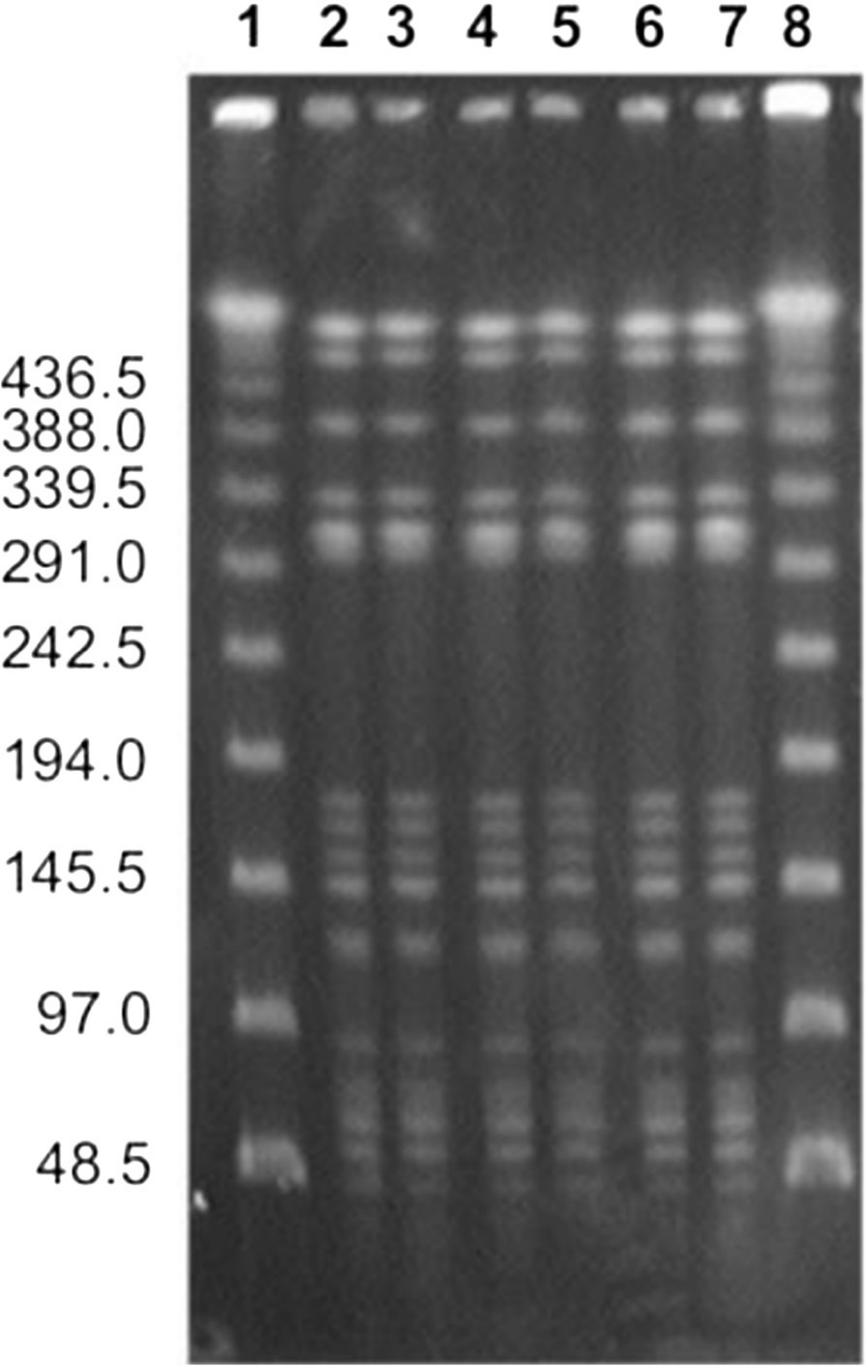
**PFGE profiles of the *bla***_**NDM4**_ -**positive *E.coli *isolates following digestion with XbaI.**

*E. coli* can be classified as phylogroup A, B1, B2 or D according to the phylogenetic relationship of the sequences. Phylogenetic analysis showed that isolates belonged to the phylogenetic group D, which includes extra-intestinal isolate. All isolates exhibited the same PFGE macrorestriction profile (Figure 2).

### MLST

All the NDM4-positive isolates were designated to a certain MLST sequence type by the combination of the seven allelic housekeeping genes. MLST analysis revealed that all isolates belonged to sequence type 405 (ST405).

### Genetic context of *bla*_NDM4_

**Figure 3:**
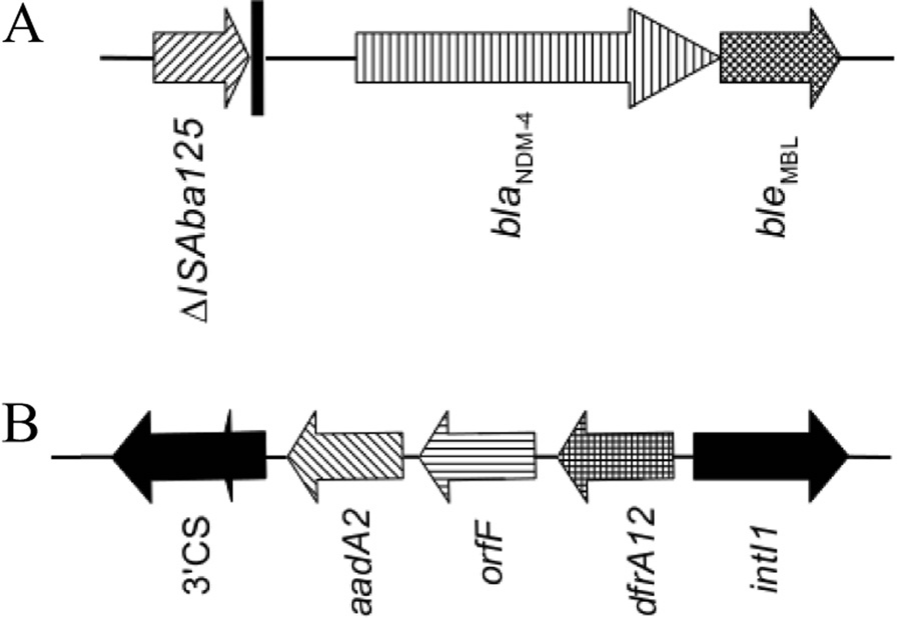
**Schematic representation of genetic structures surrounding *bla***_**NDM4**_** (A) and structure of class 1 integron (B).**

In the index isolate, PCR and sequencing analysis detected the presence of *bla*_NDM-4_ and of the following acquired resistance genes: *bla*_TEM-1_, *bla*_CTX-M-15,_*dfrA12*, *aac* (*3*)-*II*, *aadA2*. No other carbapenemase genes (OXA-48 or VIM types) were identified in these isolate. The resistance determinants *dfrA12* and *aadA2* were carried on gene cassette inserted into a class 1 integron (Figure 3), resulting in a cassette array identical to that previously described in *E.coli* GUE-NDM isolate from India (accession number JQ364967).

Genetic structures surrounding the *bla*_NDM-4_ gene performed by PCR identified immediately upstream of the gene the IS*Aba125* insertion sequence and downstream of the gene was identified the *ble*_MBL_ gene encoding the resistance to bleomycin (Figure 3).

### Plasmid features

The *bla*_NDM_ gene could not be transferred by conjugation to *E.coli* J53 recipient. All strains carried a large plasmid (>23 Kb) and when the plasmid band was extracted from the gel and used as templates for the amplification of the *bla*_NDM_ and *bla*_CTX-M_ genes, the specific products were detected, suggesting that both resistance determinants resided in this plasmid. The PCR-based replicon typing method showed that *bla*_NDM-4_ -positive plasmid belonged to the IncF incompatibility group.

## Discussion

In this communication, we described the first isolation of NDM-4 producing *E.coli* in Italy, represented by *E.coli* of sequence type 405(ST405). *E.coli* ST405 belonging to phylogenetic group D is increasingly reported as multidrug resistant strains causing extra-intestinal infections [20] and is a well-known pandemic clonal lineage implicated as vehicles driving the international spread of *bla*_*CTX-M*_[21].

NDM is not associated with certain clones, plasmids or transposons [13], our *bla*_NDM-4_ -positive plasmid belonged to the IncF incompatibility group which is known to be a major vehicle for dissemination of the *bla*_CTX-M-15_ gene [22].

By analyzing genetic structures surrounding the *bla*_NDM-4_ gene, we identified insertion sequence IS*Aba125* upstream and the bleomycin resistance gene downstream of the *bla*_NDM-4_ gene, this genetic environment of our NDM isolates was the same observed for most NDM-1 positive enterobacterial isolates [13].

In both patients, after treatment with in *vitro* active antimicrobial agents (colistin and nitrofurantoin), clinical improvement was observed and in subsequent urine samples of patient 1 *E. coli* NDM-4 was no longer isolated. Patient 2 was discharged without further microbiological investigation.

Patient 1 was previously hospitalized in India, a geographical region with high prevalence of NDM-producing isolates. This is the first example of importation of an Indian NDM-4-producing isolate in Italy following a hospital transfer, confirming the recent observations suggesting that the Indian subcontinent may represent an important reservoir of NDM producers. Because patient 2 had not a history of travel to NDM endemical areas and PFGE profile of the strains was identical, it is plausible that a spread of NDM-4 -producing *E.coli* from patient 1 to patient 2 occurred.

According to the hospital microbiology laboratory records, no further isolation of NDM-4-positive bacteria was reported to date in our hospital. To our knowledge, we report here the first NDM-4 producing *E.coli* detected in Italy and the fourth worldwide [2,3,23] . NDM-4 producing *E.coli* strains have been previously described in patients from India, Cameroon and Denmark. In this last case, the Danish patient was previously hospitalizes in Vietnam. In three cases (Cameroon, Denmark and Italy), isolates belonged to the ST405 sequence type. This finding is alarming because, ST405, has been previously identified as a successful international sequence type and it could favor the spread of NDM producers.

## Conclusions

This is the first report on the emergence of an MDR strain of *E.coli* producing the NDM-4 MBL in Italy as the result of importation of an Indian NDM-4-producing isolate following a hospital transfer. The isolate belonged to a well-known international sequence type (ST405) able to spread and cause outbreak.

Our data confirms the need for a systematic screening to rapidly detect NDM-producing strains especially among patients previously hospitalized in the endemic geographic areas to avoid dissemination of carbapenemase-producing Enterobacteriaceae.

## Abbreviations

NDM: New Dehli Metallo-beta-Lactamase; MBL: Metallo-beta-Lactamase; PFGE: Pulse-Field Gel Electrophoresis; PCR: Polymerase Chain Reaction; MDR: Multidrug resistant; EUCAST: European Committee on Antimicrobial Susceptibility Testing; MLST: Multi Locus Sequence Typing; MIC: Minimum Inhibitory Concentration.

## Competing interests

The authors declare that they have no competing interests.

## Authors’ contributions

EC carry out the experiments AM carried out microbiological diagnostic analysis, designed the study and wrote the manuscript; FV, VDB and MC produced clinical and infectious diseases data and revised the manuscript, GO implemented microbiological procedures to detect carbapenemase producing strains and monitored their emergence during the study period. CV critically revised the manuscript. All authors read and approved the final version for publication.

## Authors’ information

Erika Coppo is a Microbiology PhD student working at the Microbiology Unit, DISC, University of Genoa, Italy. Valerio Del Bono, MD, has been working since 1994 in Infectious Disease department in Genoa as attending physician in chief. He is a member, as a responsible for Infectious Disease, of the healthcare-associated infection control team of San Martino-IST Hospital. He acts as a referee for several international journals. He is author or co-author of more 40 internationally published papers. Francesco Ventura is the head of the rehabilitation Unit, IRCCS AOU San Martino-IST, Genoa. Marco Camera is a specialist in Infectious Disease working at the IRCCS AOU San Martino-IST, Genoa Giovanni Orengo was medical director of San Martino-IST Hospital until 2011 and then director of hospital hygiene to date. His main goal was to implement active microbiological surveillance systems and he’s director of the Committee for the fight against Nosocomial Infections. Claudio Viscoli is Full Professor of Infectious Diseases at the University of Genoa, Genoa, Italy. He is the head of the Infectious Diseases Unit, IRCCS AOU San Martino-IST, Genoa. He had published more than 100 international papers. Anna Marchese is Associate Professor of Clinical Microbiology at the University of Genoa, Genoa, Italy. Her research fields include: epidemiology of mechanisms of antibiotic resistance, antimicrobial susceptibility testing, antimicrobial profile of new drugs, bacterial genetics. She has published more than 80 international papers.

## References

[B1] Bonomo RA New Delhi metallo-beta-lactamase and multidrug resistance: a global SOS? Clin Infect Dis 2011 52 485 487 10.1093/cid/ciq179 21258101

[B2] Nordmann P Boulanger AE Poirel L NDM-4 metallo-β-lactamase with increased carbapenemase activity from *Escherichia coli* Antimicrob Agents Chemother 2012 56 2184 2186 10.1128/AAC.05961-11 22252797 PMC3318389

[B3] Dortet L Poirel L Anguel N Nordmann P New Dehli metallo- β-lactamase 4-producing *Escherichia coli* in Cameroon Emerg Infect Dis 2012 18 1540 1542 10.3201/eid1809.120011 22932298 PMC3437724

[B4] D’Andrea MM Venturelli C Giani T Arena F Conte V Bresciani P Rumpianesi F Pantosti A Narni F Rossolini GM Persistent carriage and infection by multidrug-resistant *Escherichia coli* ST405 producing NDM-1 carbapenemase: report on the first italian cases J Clin Microbiol 2011 49 Suppl 7 2755 2758 21525229 10.1128/JCM.00016-11 PMC3147842

[B5] Gaibani P Ambretti S Berlingeri A Cordovana M Farruggia P Panico M Landini MP Sambri V Outbreak of NDM-1-producing *Enterobacteriaceae* in northen Italy, July to August 2011 Euro Surveill 2011 16 Suppl 47 20027 22152705

[B6] The European Committee on Antimicrobial susceptibility testing Breakpoint tables for interpretation of MIC’s and zone diameters 2014 Version 4.0, 2014. http://www.eucast.org

[B7] Arakawa Y Shibata N Shibayama K Kurokawa H Yagi T Fujiwara H Goto M Convenient test for screening metallo-β-lactamase-producing gram-negative bacteria by using thiol compounds J Clin Microbiol 2000 38 40 43 10618060 10.1128/jcm.38.1.40-43.2000 PMC86013

[B8] Yuan M Aucken H Hall LM Pitt TL Livermore DM Epidemiological typing of Klebsiella with extended-spectrum β-lactamases from European intensive care units J Antimicrob Chemother 1998 41 527 539 10.1093/jac/41.5.527 9630406

[B9] Tenover FC Arbeit RD Goering RV Mickelsen PA Murray BE Persing DH Swaminathan B Interpreting chromosomal DNA restriction patterns produced by pulsed-field gel electrophoresis: criteria for bacterial strain typing J Clin Microbiol 1995 33 2233 2239 7494007 10.1128/jcm.33.9.2233-2239.1995 PMC228385

[B10] Hornsey M Phee L Wareham DW A novel variant, NDM-5, of New Delhi metallo beta lactamase in a multidrug resistant *Escherichia coli* ST648 isolate recovered from a patient in the United Kingdom Antimicrob Agents Chemother 2011 55 Suppl 12 5952 5954 21930874 10.1128/AAC.05108-11 PMC3232805

[B11] Pagani L Dell’Amico E Migliavacca R D’Andrea MM Giacobone E Amicosante G Romero E Rossolini GM Multiple CTX-M-type extended-spectrum beta-lactamases in nosocomial isolates of Enterobacteriaceae from a hospital in northern Italy J Clin Microbiol 2003 41 Suppl 9 4264 4269 12958255 10.1128/JCM.41.9.4264-4269.2003 PMC193787

[B12] Sáenz Y Briñas L Domínguez E Ruiz J Zarazaga M Vila J Torres C Mechanisms of resistance in multiple-antibiotic-resistant *Escherichia coli* strains of human, animal, and food origins Antimicrob Agents Chemother 2004 48 Suppl 10 3996 4001 15388464 10.1128/AAC.48.10.3996-4001.2004 PMC521888

[B13] Poirel L Dortet L Bernabeu S Nordmann P Genetic features of *bla*_NDM-1_-positive Enterobacteriaceae Antimicrob Agents Chemother 2011 54 (Suppl 11) 5403 5407 21859933 10.1128/AAC.00585-11 PMC3195013

[B14] Clermont O Bonacorsi S Bingen E Rapid and sample determination of the *Escherichia coli* phylogenetic group Appl Environ Microbiol 2000 66 4555 4558 10.1128/AEM.66.10.4555-4558.2000 11010916 PMC92342

[B15] Poirel L Lagrutta E Taylor P Pham J Nordmann P Emergence of metallo-β-lactamase NDM-1-producing multidrug-resistant *Escherichia coli* in Australia Antimicrob Agents Chemother 2010 54 4914 4916 10.1128/AAC.00878-10 20823289 PMC2976126

[B16] Lévesque C Roy P Smith DHTF, Tenover FC, White TJ PCR analysis of integrons, p. 590–594 In Persing Diagnostic molecular microbiology:principles and application 1993 Washington, DC: American Society for Microbiology

[B17] Lauretti L Riccio ML Mazzariol A Cornaglia G Amicosante G Fontana R Rossolini GM Cloning and characterization of bla_VIM_, a new integron-borne metallo-beta-lactamase gene from a *Pseudomonas aeruginosa* clinical isolate Antimicrob Agents Chemother 1999 43 Suppl 7 1584 1590 10390207 10.1128/aac.43.7.1584 PMC89328

[B18] Tokatlidou D Tsivitanidou M Pournaras S Ikonomidis A Tsakris A Sofianou D Outbreak caused by a multidrug-resistant *Klebsiella pneumoniae* clone carrying *bla*_VIM-12_ in a University hospital J Clin Microbiol 2008 46 Suppl 3 1005 1008 18199780 10.1128/JCM.01573-07 PMC2268336

[B19] Carattoli A Bertini A Villa L Falbo V Hopkins KL Threlfall EJ Identification of plasmids by PCR based replicon typing J Microbiol Methods 2005 63 219 228 10.1016/j.mimet.2005.03.018 15935499

[B20] Novais A Vuotto C Pires J Montenegro C Donelli G Coque TM Peixe L Diversity and biofilm-production ability among isolates of *Escherichia coli* phylogroup D belonging to ST69, ST393 and ST405 clonal groups BMC Microbiol 2013 13 144 10.1186/1471-2180-13-144 23800205 PMC3695789

[B21] Coque TM Novais A Carattoli A Poirel L Pitout J Peixe L Baquero F Cantón R Nordmann P Dissemination of clonally related *Escherichia coli* strains expressing extended-spectrum beta-lactamase CTX-M-15 Emerg Infect Dis 2008 14 Suppl 2 195 200 18258110 10.3201/eid1402.070350 PMC2600198

[B22] Boyd DA Tyler S Christianson S McGeer A Muller MP Willey BM Bryce E Gardam M Nordmann P Mulvey MR Complete nucleotide sequence of a 92-kilobase plasmid harboring the CTX-M-15 extended-spectrum beta-lactamase involved in an outbreak in long-term-care facilities in Toronto, Canada Antimicrob Agents Chemother 2004 48 Suppl 10 3758 3764 15388431 10.1128/AAC.48.10.3758-3764.2004 PMC521865

[B23] Jakobsen L Hammerum AM Hansen F Fuglsang-Damgaard D An ST405 NDM-4-producing *Escherichia coli* isolated from a Danish patient previously hospitalized in Vietnam J Antimicrob Chemother 2014 69 Suppl 2 559 560 24013194 10.1093/jac/dkt356

